# HLA-G expression associates with immune evasion muscle-invasive urothelial cancer and drives prognostic relevance

**DOI:** 10.3389/fimmu.2024.1478196

**Published:** 2024-10-14

**Authors:** Annalena Branz, Christian Matek, Fabienne Lange, Veronika Bahlinger, Niklas Klümper, Michael Hölzel, Pamela L. Strissel, Reiner Strick, Danijel Sikic, Sven Wach, Helge Taubert, Bernd Wullich, Arndt Hartmann, Barbara Seliger, Markus Eckstein

**Affiliations:** ^1^ Institute of Pathology, Universitätsklinikum Erlangen, Friedrich-Alexander-Universität Erlangen-Nürnberg (FAU)), Erlangen, Germany; ^2^ CCC Erlangen-EMN: Comprehensive Cancer Center Erlangen-EMN (CCC ER-EMN), Erlangen, Germany; ^3^ CCC WERA: Comprehensive Cancer Center Alliance WERA (CCC WERA), Erlangen, Germany; ^4^ BZKF: Bavarian Cancer Research Center (BZKF), Erlangen, Germany; ^5^ Department of Pathology and Neuropathology, University Hospital and Comprehensive Cancer Center Tübingen, Tübingen, Germany; ^6^ Department of Urology and Pediatric Urology, University Hospital Bonn, Bonn, Germany; ^7^ Institute of Experimental Oncology, University Medical Center Bonn (UKB), Bonn, Germany; ^8^ Center for Integrated Oncology Aachen/Bonn/Cologne/Düsseldorf (CIO-ABCD), Bonn, Germany; ^9^ Department of Gynecology and Obstetrics, Universitätsklinikum Erlangen, Friedrich-Alexander-Universität Erlangen-Nürnberg (FAU), Erlangen, Germany; ^10^ Department of Urology and Pediatric Urology, Universitätsklinikum Erlangen, Friedrich-Alexander-Universität Erlangen-Nürnberg (FAU), Erlangen, Germany; ^11^ Institute of Translational Immunology, Medical School Brandenburg, Brandenburg, Germany; ^12^ Medical Faculty, Martin Luther University Halle-Wittenberg, Halle, Germany

**Keywords:** HLA-G, urothelial cancer, immune checkpoints, immune evasion, immune microenvironment

## Abstract

**Introduction:**

Urothelial bladder cancer is frequent and exhibits diverse prognoses influenced by molecular subtypes, urothelial subtype histology, and immune microenvironments. HLA-G, known for immune regulation, displays significant membranous expression in tumor tissues.

**Methods:**

We studied the protein expression of Human Leucocyte Antigen G (HLA-G) in 241 Muscle-Invasive Bladder Cancer (MIBC) patients, elucidating its potential clinical and biological significance. Protein expression levels were evaluated and correlated with molecular subtypes, histological characteristics, immune microenvironment markers, and survival outcomes.

**Results:**

High HLA-G expression associates with poor overall survival (OS) and diseasespecific survival (DSS), independent of clinicopathological parameters. HLA-G expression varies among molecular subtypes and Urothelial Subtype Histology, e.g., elevated expression levels in basal/squamous MIBC and those with sarcomatoid differentiation. Notably, HLA-G is increased in MIBC with an immune evasive microenvironment (high PD-L1 tumor cell expression, NK cell depletion, granzyme B (GZMB)/CD8 ratio reduction, MHC class I (MHCI) expression reduction) that are characterized by immunosuppressive features and poor prognosis. Furthermore, HLA-G correlates with elevated levels of other immune checkpoint proteins (TIGIT, LAG3, CTLA-4), indicating its role in immune evasion.

**Discussion:**

Our findings underscore HLA-G’s role as a potential prognostic marker and interesting immunotherapeutic target in MIBC. Its impact on immune evasion mechanisms and broad expression, coupled with associations withpoor survival and distinct tumor phenotypes, positions HLA-G as a promising protein for further exploration in developing targeted immunotherapies for MIBC patients.

## 1 Introduction

Urothelial bladder cancer is among the 10 most common tumor entity in industrialized countries. While 75% are non-invasive tumors with a good patients’ prognosis, the remaining 25% are invasive and still associated with poor outcomes (1). The standard of care for muscle-invasive bladder cancer (MIBC) is currently radical cystectomy with perioperative platinum-based chemotherapy and adjuvant nivolumab treatment in selected cases (2). Molecular analyses identified three major molecular lineages within muscle-invasive urothelial bladder cancer (MIBC): basal, luminal, and neuronal-like/neuroendocrine. These subtypes have considerable prognostic and predictive implications (3–8) and correlate with urothelial subtype histology (9). Moreover, the basal and luminal subtypes of MIBC exhibit distinct associations with inflammation levels, infiltration through T lymphocytes, and secondary lymphoid structures, which are valuable immunological indicators predicting survival outcomes and therapy responses of patients (10–13).

Human leukocyte antigen G (HLA-G) is known for its immune regulatory properties in the placenta to prevent fetal rejection in pregnant women (14–16) and (aberrantly) expressed in many tumor entities and at immune-privileged sites to induce immune tolerance (17–21). HLA-G binds to the inhibitory ligands ILT2, ILT4, and KIR2DL4 and suppresses effector mechanisms, in particular in CD8+ T cell and NK cell mediated cytotoxicity, thereby contributing to the immune escape of tumors (18, 22). The tumor-specific expression of HLA-G and its biological characteristics, particularly its involvement in tumor immune evasion, render it as an interesting biomarker and a potential therapeutic target for novel immunotherapies. However, to date, the expression of HLA-G in MIBC remains unexplored. Thus, the global expression patterns and association of HLA-G with determinants of the spatial immune microenvironment, molecular subtypes and pathological features was analyzed in the well characterized consecutive population-based CCC-EMN-ER MIBC patient cohort (n=241).

## 2 Methods

### 2.1 Study cohort

Clinical and pathological data and formalin-fixed paraffin-embedded (FFPE) specimens used for immunohistochemical analysis were obtained through a retrospective (cases 2002-2016) and a prospective (from 2016) review of the medical records of 241 patients diagnosed with and treated for MIBC at the University Hospital Erlangen from 2002 to 2020 (11, 23). All patients were treated with radical cystectomy and bilateral lymph node dissection for curative intent; selected patients with pT-stage pT3 or higher or/and lymph node metastasis received adjuvant platinum-containing chemotherapy (n=57). None of the patients received neoadjuvant chemotherapy. The clinicopathological characteristics of the study cohort are summarized in **Supplementary Table 1**. The median overall survival (OS) of patients after surgery was 29.5 months [95%-CI=20.4-38.5]. All carcinomas were pathologically re-examined and classified according to the World Health Organization (WHO) classification of 2022 he Union for International Cancer Control (UICC) TNM classification by two experienced uropathologists [M.E., H.A.] (9, 24). Consensus molecular subtypes (6, 25), immune phenotypes (11), and urothelial subtype histology labels were adapted from previous publications of our group (26). Standard FFPE tumor tissues were consecutively collected from pathologic routine case files. The blocks were stored at room temperature and for each analysis, fresh tissue cuts were taken from the blocks.

### 2.2 Molecular subtyping

RNA was isolated from formalin-fixed paraffin-embedded (FFPE) tissue samples and sequenced using the Lexogen Quant-Seq 3’ mRNA-seq Kit on a Illumina NovaSeq platform as recently described by our group in detail (25, 27). Subtype assignments were adapted from our previous publication (27).

### 2.3 Tissue microarray and immunohistochemistry

Hematoxylin and Eosin (HE) stained slides were scanned and four tissue cores (diameter 1.6 mm, two of the invasion front, two of the tumor center) were punched out of donor FFPE blocks using the TMA-Gandmaster (3DHistech, Hungary) and transferred in a 70-place recipient multiblock. Immunohistochemical stains were performed on 4 µm TMA tissue sections using a Ventana Benchmark Ultra autostainer (Ventana) for the following proteins: HLA-G, MHCI ABC, LAG3, CTLA-4, CD8, CD68, PD-1, CD56, PD­L1, FOXP3, GZMB. Details on the staining protocols are provided in **Supplementary Table 2**. The HLA-G antibody recognizes the amino acids 61–83 of the α-1 domain, that is present in all isotypes of HLA-G, thus not allowing to discern different splicing variants of HLA-G, but the total HLA-G protein expression.

### 2.4 Evaluation of immune cell populations, PD-L1, MHC-I and HLA-G

HLA-G and MHC-I ABC expression was determined by estimating the percentage of tumor cells positive for membrane staining (0-100%) and the relative intensity of membranous expression (0-3+), and subsequently quantified using the well-established H-score algorithm ranging from 0 (negative) to 300 (100% of cells positive with 3+ intensity). In detail, the H-score is calculated by the percentage of HLA-G high expressing tumor cells * 3 plus percentage of HLA-G intermediate expressing tumor cells * 2 plus percentage of HLA-G low expressing tumor cells *1. The H-score of two tumor center cores and two invasion front cores was collected and for subsequent analyses the mean value was calculated. PD-L1 was assessed via the combined positivity score (CPS) integrating the relative amount of PD-L1 expressing tumor associated immune cells and PD-L1 positive tumor cells related to all present tumor cells as described in detail previously (28). Immune cells expressing LAG3, CTLA-4, CD8, CD68, PD-1, CD56, FOXP3, and GZMB were quantified digitally as positive cells per mm2 tumor tissue area at the tumor center and invasion front (analyzed regions of interest were annotated by an experienced pathologist, M.E.); for subsequent analyses the mean value was calculated from different measurements obtained from the tumor center and invasion front. Digital immune cell quantification was performed using the open source software QuPath v0.4.4., i.e., the positive cell quantification platform (29).

### 2.5 Statistical analysis

OS and disease-specific survival (DSS; time to disease-specific death) curves were estimated by Kaplan–Meier regressions. Multivariable survival analyses were conducted using Cox proportional hazards regression modeling to assess the magnitude of impact while adjusting for clinicopathological parameters (sex, age, pT-Stage, pN-Stage, WHO 1973 grading, WHO 2016 grading, lymphovascular invasion, blood vessel invasion, perineural invasion, receipt of adjuvant platinum-based chemotherapy, immune phenotype, urothelial subtype histology). Test for normal distribution was performed by Shapiro-Wilk test. Multiple group comparisons were carried out using non-parametric Kruskal-Wallis tests and Dunn´s multiple comparisons test. Correlation analysis was performed by Spearman´s non-parametric test. All statistical analyses were performed by GraphPad Prism 8.3.0 (GraphPad Software Inc., La Jolla, CA, USA) and R-Studio (v2022.07.1 + 554; Rv4.2.1). All *P*-values were two-sided, and *P*-values < 0.05 were considered significant.

### 2.6 Ethics

The present study was approved by the FAU ethical review board 97_18 Bc, 22-343-B in accordance with the Declaration of Helsinki. All patients gave written informed consent.

## 3 Results

### 3.1 HLA-G expression in MIBC

**Figure 1:**
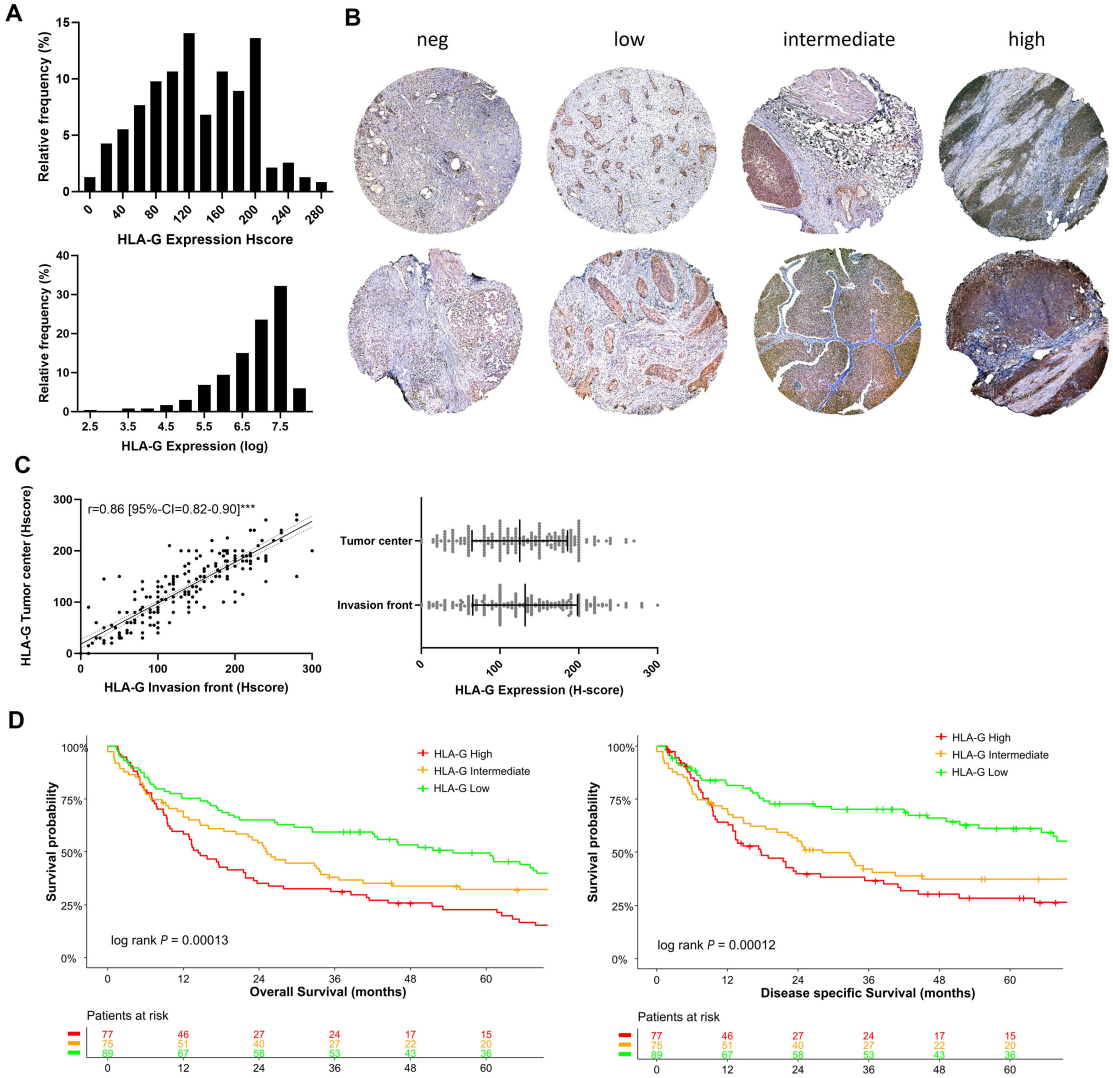
HLA-G expression patterns in MIBC: **(A)** Histogram of HLA-G expression in H-score scale and logarithmic scale. The Shapiro-Wilk test indicates that the expression of HLA-G is not normally distributed (*P*=0.01, significance level of 0.05). **(B)** Expression levels of HLA-G at the invasion front (upper row) and tumor center (lower row) in MIBC. **(C)** Spearman rank correlation and not significant Mann-Whitney test (*P*=0.30) of HLA-G expression of tumor center and evasion front. **(D)** Kaplan-Meier analysis of overall (OS) and disease specific survival (DSS) based on HLA-G expression levels (low, intermediate, high). Univariable log-rank *P* value is depicted in the lower left corner of the survival plots. Table shows the number of patients at risk in 12 months increments. Statistical significance at *P*<0.05; ***significance at *P*<0.0001.

In our cohort of 241 patients, most tumors showed a membranous HLA-G expression with an H-score median of 125 and an interquartile range of 80-175 (**Figure 1A**), whereas surrounding stromal tissues lacked HLA-G expression (internal negative control). **Figure 1B** shows representative samples of different HLA-G expression levels (negative, low, intermediate, high) at the tumor center and the invasion front. HLA-G showed a similar expression pattern at the tumor center and the invasion front without significant differences in expression levels (Spearman correlation coefficient r=0.86 [95%-CI=0.82-0.90]; **Figure 1C**). A Shapiro-Wilk test performed on HLA-G distribution indicated that HLA-G protein expression is not normally distributed in our cohort which could be attributed to the size of the cohort (*P*=0.01, significance level threshold *P*<0.05).

### 3.2 HLA-G expression is associated with poor overall survival

The tumors were divided into three subgroups depending on the HLA-G expression levels with cut-offs set by objective tertile splitting resulting in three expression groups (“HLA-G High”, “HLA-G Intermediate”, “HLA-G Low”). In univariable Kpalan-Meier analysis high HLA-G expression was associated with significantly decreased median OS rates of 14.3 months (95%-CI=10.2-23.1) versus 54.6 months (95%-CI=41.9-79.9) in patients with “HLA-G high” versus “HLA-G low” tumors (log Rank *P*<0.001; **Figure 1D**). “HLA-G high” expression status remained as an independent predictor for worse OS in a multivariable Cox Regression model (OS: HLA-G low ~ HLA-G high HR: 2.41 [95%-CI=1.57-3.70]; **Supplementary Figure 1**). In contrast to HLA-G protein expression on tumor cells, HLA-G expression detected on tumor infiltrating lymphocytes (TILs) was not associated with patient survival rates (**Supplementary Figure 2**).

### 3.3 Association of HLA-G protein expression with molecular subtypes and urothelial subtype histology

**Figure 2:**
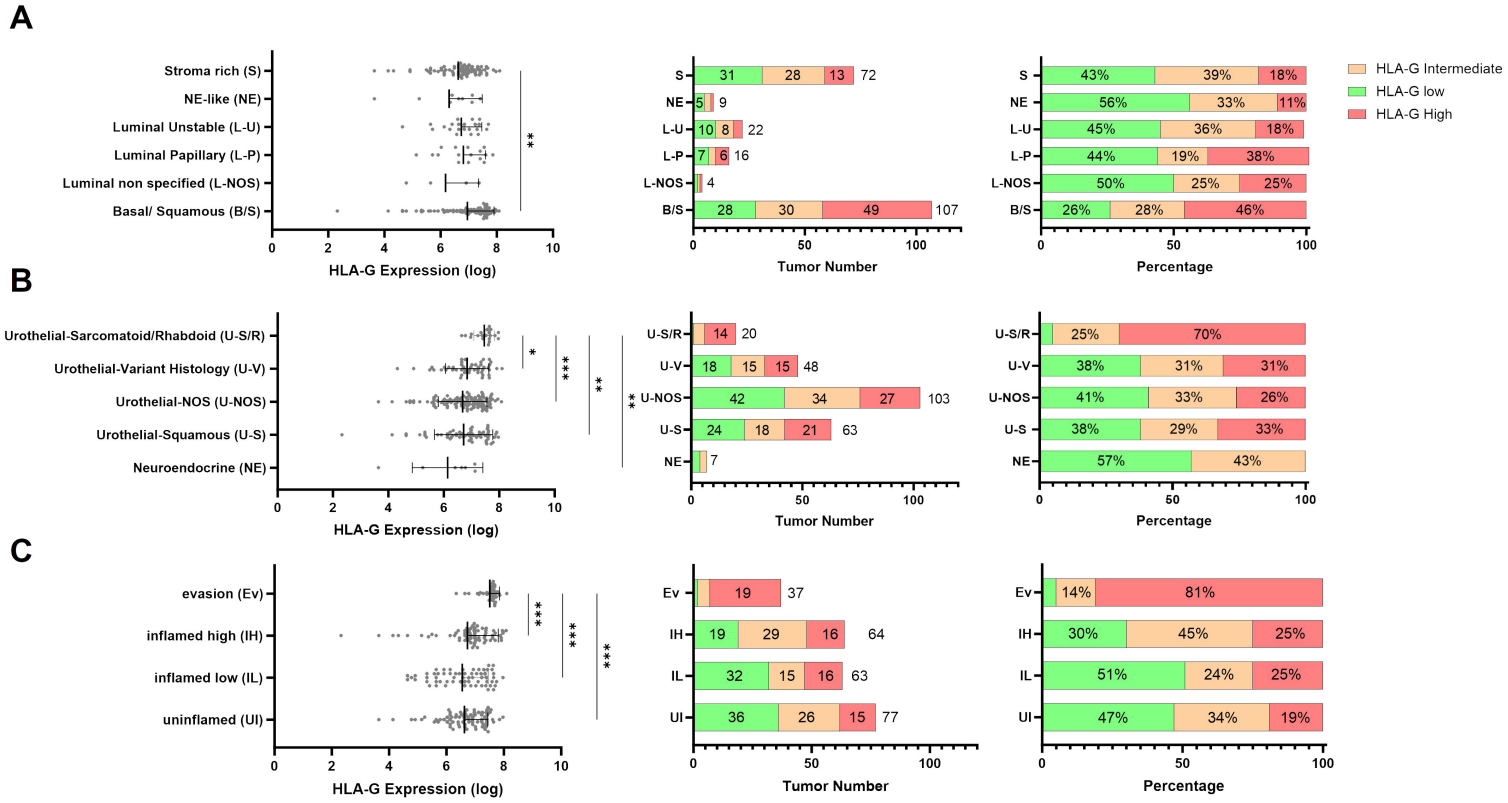
Distribution of continuous HLA-G expression by **(A)** molecular subtypes (n=230), **(B)** urothelial subtype histology (n=241), and **(C)** immune phenotypes (n=241). *P* values are derived by Dunn´s multiple comparisons test. Distribution of HLA-G low, intermediate, and high expressing tumors in **(A)** Urothelial Subtype Histology, **(B)** Molecular subtypes, and **(C)** immune phenotypes in absolute number and percentage. Statistical significance at *P*<0.05; *significance at *P*<0.01; **significance at *P*<0.001; ***significance at *P*<0.0001.

To analyze whether HLA-G expression is dependent on MIBC molecular consensus subtypes, that have been defined by Kamoun et al. (6) we correlated HLA-G protein expression on tumor cells with different molecular consensus subtype classes that have been called from whole transcriptome sequencing data from our MIBC cohort using the consensusMIBC R package (https://github.com/cit-bioinfo/consensusMIBC). We found that tumors with basal squamous (Ba/Sq) molecular subtype showed significantly higher HLA-G protein expression on tumor cells compared to tumors with luminal, stroma-rich or neuroendocrine-like molecular subtypes (Kruskal-Wallis test for multiple group comparisons: *P*=0.004; **Figure 2A**). The single group comparison *P*-values are summarized in **Supplementary Table 3C**.

Furthermore, MIBC with sarcomatoid/rhabdoid morphology, that are often Ba/Sq and known to exhibit strong epithelial to mesenchymal transition (EMT) activity and usually present with advanced pT-stage and increased metastatic incidence at the time of diagnosis (30–32), exhibited the highest HLA-G expression levels on tumor cells (Kruskal-Wallis test for multiple group comparisons: *P*=0.0003; **Figure 2B**). The single group comparison *P*-values are summarized in **Supplementary Table 3D**.

### 3.4 High HLA-G expression levels associated with an immunosuppressive microenvironment in MIBCs

**Figure 3:**
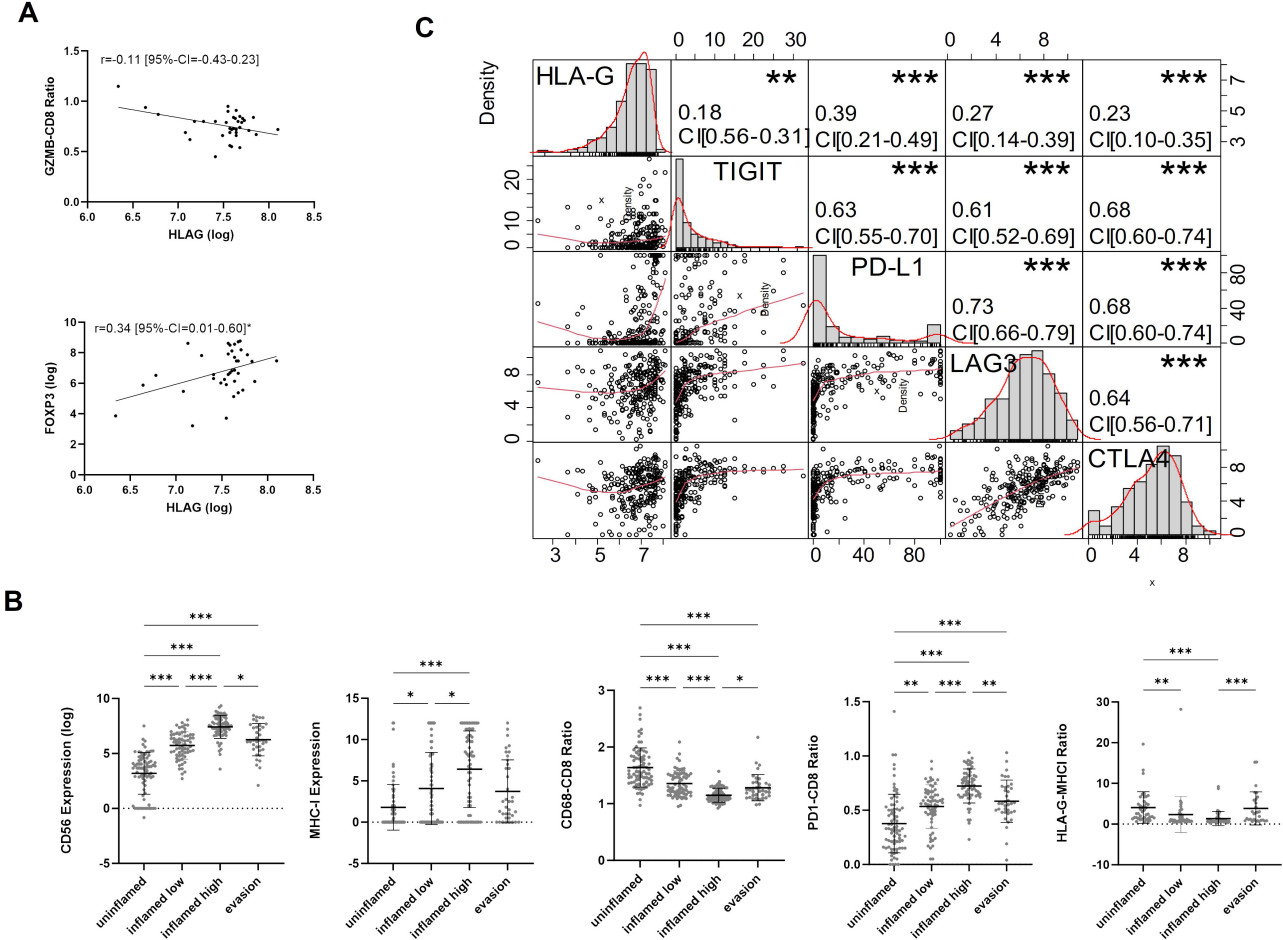
**(A)** Spearman-rank Correlation of HLA-G expression and GZMB-CD8 ratio and FOXP3 expression in the evasion phenotype. **(B)** Characterization of TIME (CD56, MHCI, CD68-CD8 Ratio, PD1-CD8 ratio, HLA-G-MHCI Ratio) across immune phenotypes. P-values are derived by Dunn´s multiple comparisons test. **(C)** Correlation matrix for pairwise associations between ICP protein expression. Correlation plots are presented in the bottom left part, histograms of logarithmic protein expression levels in the diagonal, the corresponding absolute correlation coefficients, and the level of statistical significance (Spearman correlation test) in the upper right part. Statistical significance at *P*<0.05; *significance at *P*<0.01; **significance at *P*<0.001; ***significance at *P*<0.0001.

To further investigate the immunological features of HLA-G expressing or negative tumors we correlated HLA-G protein expression with spatially organized MIBC immune phenotypes that were described by *Pfannstiel et al.* using a hierarchical cluster analysis based on T-cell, NK-cell, and macrophage infiltration as well as the distribution of PD-L1 expression on immune and tumor cells (11). HLA-G was particularly highly expressed in tumors characterized as “evasion phenotypes” (**Figure 2C**, Kruskal-Wallis test for multiple group comparisons: *P*<0.0001, single group *P*-values in **Supplementary Table 3E**). There was no significant difference in HLA-G expression between the other immune phenotypes in a single group comparison (**Supplementary Table 3E**). Among 37 tumors of the evasion phenotype, 28 had a basal/squamous molecular subtype and seven showed a sarcomatoid/rhabdoid morphology matching with our above observation of higher HLA-G protein expression in MIBCs with sarcomatoid histology and Ba/Sq subtypes (**Supplementary Figure 3B**) (6). To investigate what distinguishes the evasion phenotype with its high HLA-G expression from other immunee phenotypes, infiltration with FOXP3 positive regulatory T cells (33) and the GZMB – CD8 ratio- as an indicator for a defective T cell effector function- were determined in the evasion phenotype subset (34, 35). Strikingly, HLA-G significantly and positively correlated with elevated FOXP3+ Treg infiltration (**Figure 3A**: Spearman correlation coefficient r=0.340*) and negatively with the GZMB - CD8 ratio (**Figure 3A**: Spearman correlation coefficient r=-0.107), indicating a increased immunosuppression by Treg infiltration and an impaired cytotoxicity by higher prevalence of GZMB-depleted CD8+ cytotoxic T-cells. Matching with these observations, we also found a lower CD56+ NK cell infiltration in the evasion phenotype compared with the inflamed high phenotype (*P*=0.012), as well as a more myeloid weighted immune infiltrate with a higher CD68/CD8 ratio and a lower a lower PD-1-CD8 ratio indicating less activated T-cells compared to the inflamed high phenotype (*P* =0.043) (**Figure 3B**).

### 3.5 HLA-G is co-expressed with other immune checkpoints

Correlation analysis between HLA-G and immune checkpoint proteins TIGIT, PD-L1 CPS (combined positive score), LAG3, and CTLA4 revealed significant positive correlations with Spearman’s correlation coefficients amounting 0.18 (95%-CI=0.06-0.31), 0.39 (95%-CI=0.27–0.49), 0.27 (95%-CI=0.14–0.39), and 0.23, respectively (**Figure 3C**). All those markers are expressed in exhausted T cells and are considered to be involved in a defective anti-tumor immune response, thus indicating a potential immunosuppressive infleunce of HLA-G tumor cell expression (36, 37).

## 4 Discussion

MIBC continues to exhibit a poor prognosis with a 5-year survival of 40%-60% (38). The immune system plays an important role in the anti-tumour response and immune-targeted therapies, such as immune checkpoint blockade have already been tested and successfully implemented for MIBC and metastatic UC treatment (39). However, the development of future immunotherapies requires a deeper understanding of the tumor immune microenvironment (TIME) and immune evasion mechanisms of MIBC. Prior research has demonstrated an increase in HLA-G expression across diverse tumor entities, which is associated with a poor patients’ prognosis (21, 40). Systematic reports on the prevalence and biological relevance of HLA-G in MIBC however are missing until today. Thus, we conducted the present study to better understand if and which role HLA-G play in the clinical and biological framework of MIBC with a specific focus on elucidating its role in MIBC immune evasion, and thus studying HLA-G as a potential therapeutic target for restoring the anti-tumoral immunity.

Based on our findings, HLA-G appears to be a promising biomarker associated with the biological characteristics, the composition of the tumor immune microenvironment (TIME), and clinical outcomes in MIBC. This is supported by the observation that patients with high HLA-G expression in tumors experience reduced overall and disease-specific survival. Additionally, the ability to quantify soluble HLA-G in the blood, its predictive value when co-expressed with other immune checkpoints, and its consistent presence across various tumor regions further highlight its utility as a potential biomarker and therapeutic target. However, our study is limited by assessing total HLA-G expression while a functional heterogeneity of HLA-G isoforms exists, and the retrospective and monocentric nature of the study.

Our data demonstrated a strong correlation between HLA-G expression on tumor cells and both overall survival and disease specific survival rates in MIBC patients, highlighting the clinical and biological significance of HLA-G in cancer progression and prognosis. In contrast, HLA-G expression on TILs was not linked to a decrease in survival rates, suggesting that it is HLA-G expression on tumour cells, rather than on TILs, that orchestrates biological behavior of MIBC and thus HLA-G’s impact on clinical outcomes. Notably, the impact of HLA-G tumor cell expression on overall and disease specific survival remained independent of clinicopathological parameters in the multivariable regression analysis. This is validated by recent studies which revealed a similar clinical impact of HLA-G tumor cell expression and an association with shorter overall survival in other tumor entities (40). For example, *Yie et al.* demonstrated an upregulation of HLA-G in esophageal squamous carcinomas that represents an independent adverse prognostic factor (41), and similar results were observed for non-small cell lung cancer (NSCLC) (42).

Notably, in our MIBC cohort HLA-G expression on tumor cells was highly expressed in sarcomatoid/rhabdoid tumors that show an increased EMT (43). EMT is a crucial biological process where epithelial cells differentiate into mesenchymal cells leading to increased cell mobility and invasion capacity thus facilitating metastatic seeding to other organs. Matching with these observations, EMT has been associated with poor patient outcomes, and in the case of sarcomatoid NSCLC with a remarkable upregulation of immune inhibitory molecules and immune evasion programs (44).

Hence, analogous mechanisms could be implicated in the upregulation of HLA-G in sarcomatoid MIBC. Indeed we can show that sarcomatoid MIBCs, which exhibit the highest levels of HLA-G tumor cell expression, belong predominantly to a recently described spatially organized immune phenotype (“Evasion phenotype”) whose TIME is dominated by immunosuppressive T and myeloid cells as well as a marked expression of immune inhibitory immune checkpoint molecules like PD-L1 and PD-1 on tumor cells or immune cells (11). The spatially organized immune phenotypes described by Pfannstiel et al. are also closely related to consensus molecular subtypes of MIBC, where sarcomatoid MIBCs usually belong to the basal squamous molecular subtype (Ba/Sq) (11). While MIBC with absent or low T cell infiltration mostly belong to luminal molecular subtypes, which associate with conventional urothelial histology or urothelial subtype histology, strongly T-cell infiltrated tumors predominantly belong to the basal squamous molecular subtype (Ba/Sq) and present with sarcomatoid or squamous histology (11). Matching with these observations, we also observed higher HLA-G expression levels in MIBC with Ba/Sq molecular subtypes, which is a molecular subtype that has been associated with decreased patient survival rates (6, 45). While many MIBC with Ba/Sq molecular subtypes exhibit immune infiltrates consisting of CD8+ cytotoxic T lymphocytes (CTL), NK cells and macrophages (6), especially those with high HLA-G tumor cell expression belonged to the immune evasive “Evasion phenotype” that is characterized by a T-cell exhausted and myeloid dominated TIME.

Thus, upregulation of HLA-G tumor cell expression might be a counter-regulatory mechanism that 1) limits the TIME infiltration of CD8 and NK cells, 2) exhausts present effector T cells and NK cells via recruitment of immunosuppressive myeloid cells and regulatory T-cells, and 3) increases the immunosuppressive capacity of myeloid cells (46, 47). Similar observations have been made by *Dong et al.*, where higher HLA-G expression levels was mainly found in clinically aggressive non-luminal (basal) breast cancers, and associated with depletion of activated TILs (48). Mechanistically, this immunosuppressive function of HLA-G has been partially shown to operate by inhibiting the cellular anti-tumor immune response and tumor cell killing through its interaction with receptors ILT2, ILT4, and KIR2DL4 expressed on cytotoxic T cells and NK cells (47, 49–51). In line with these observations, *Mendel et al.*, for example, demonstrated that inhibition of ILT2 (preventing HLA-G from acting via ILT2) restores antitumor immune response leading to decreased tumor growth, less metastatic spread, and a prolonged survival in a humanized mouse model (52). In addition, HLA-G has also indirect potent immune regulatory capabilities, e.g., via induction of CTLA-4 and PD-1 expression on ILT-2 positive T cells regardless of prior T cell activation (53–55). This is in line with our observations, that immune cells in the TIME of HLA-G high MIBC – especially of evasion phenotype MIBCs- exhibited an upregulation of crucial immune checkpoint proteins like TIGIT, PD-L1, LAG3, and CTLA-4 on tumor cells (PD-L1) and immune cells (all proteins). In parallel to this direct inhibition of effector T cells and NK cells, HLA-G has been linked with an active recruitment of Treg cells and induction of CD4+ T cell differentiation into Treg cells via CD46 different regulatory T cells for ex (56–58). These findings are in line with our observation of a positive correlation of regulatory T-cell infiltration and HLA-G tumor cell expression especially in MIBC with evasion phenotypes (31). Additionally, an inverse correlation between the GZMB/CD8 ratio and HLA-G was found, which is indicative of a diminished cytotoxic potential of CD8+ cytotoxic T cells (59). *Dumont et al.* associated this immunosuppressive effect on ILT2+ cytotoxic T cells to HLA-G (60), and other groups demonstrated, that this effect can be reverted effectively by inhibition of HLA-G which leads to restoration of IFNG secretion and cytotoxic degranulation (60, 61). Another frequently observed immune evasion mechanism of MIBC is the downregulation of MHC class I (MHCI) presentation on tumor cell membranes in order to evade recognition by cytotoxic T cells; however, MHC-deficient tumor cells can still be recognized and eliminated by NK cells (62–64). Interestingly, MIBC with *evasion* phenotype exhibit a low expression of MHCI accompanied by a low infiltration with CD56+ NK cells, but high levels of HLA-G expression. Thus, HLA-G upregulation might represent a mechanism to evade the MHC independent tumor cell killing through NK cells (65). This immune evasion mechanism has also been functionally proven in ovarian cancer, where HLA-G directly inhibited NK cell mediated cancer cell lysis, which could be disinhibited by blocking of HLA-G (66). Taken together, previous reports and our findings thus support the hypothesis that HLA-G is a crucial immune checkpoint protein in (subsets of) MIBC, that represents an attractive therapeutic target to restore sustained cellular anti-tumor immune response.

Beside this direct and indirect HLA-G mediated inhibition of antitumoral effector cells, recruitment and activation of myeloid cells has been functionally attributed to HLA-G (67, 68). In line with these observations, MIBC belonging to the evasion phenotype also demonstrated a myeloid-rich TIME (increased CD68/CD8 ratio) where macrophages exhibit a predominant M2 phenotype (11, 13). In line, shifts in the balance between myeloid cells and cytotoxic T cells, i.e., towards a myeloid dominated TIME, have been shown to substantially decrease adaptive antitumoral immune responses (67). In addition, HLA-G has been shown to directly increase the suppressive activity of myeloid-derived suppressor cells (MDSCs) via ILT4 in the placenta, and that this effect can be reversed by ILT4 inhibition, indicating an indirect suppression of adaptive immune responses via shaping the activity of myeloid cells (68).

Beside the HLA-G high evasion phenotype, *Pfannstiel et al., Erlmeier et al*, and *Zhou et al.* identified another subgroup of highly T cell infiltrated MIBC with a predominant infiltration by activated T cells, T cells with preserved capacity for tumor cell killing and reduced presence or absence of immune inhibitory immune checkpoint proteins (highly inflamed cytotoxic environment) as well as a reduced myeloid cell component resulting in significantly improved outcome rates of patients (11, 13, 69). In line, MIBC with this cytotoxic immune phenotype showed lower HLA-G protein expression levels than those belonging to the immunosuppressed/exhausted “Evasion phenotype” in our cohort, thus underlining the mechanistic role of HLA-G shaping the functionality of tumor associated immune infiltrates to an exhausted state. The upregulation of PD-1 on T cells within the *inflamed high* phenotype may be elucidated by the phenomenon wherein lymphocyte activation is concomitant with the upregulation of co-inhibitory receptors, thereby averting the potential for untoward inflammation in normal tissue that might result in extensive tissue damage (70).

In summary, elevated HLA-G expression is strongly linked to direct and indirect inhibition of antitumoral effector immune cells and to remodeling of the TIME to an immunosuppressive state thus resulting in significantly decreased patient survival rates. Based on previous reports and other data, we thus believe that HLA-G functions as a crucial immunosuppressive and inhibitory immune checkpoint protein in MIBC, contributing to reduced T cell cytotoxicity, suppression of NK cells and maintenance of an immunosuppressive environment. Consequently, HLA-G emerges as a promising candidate both as a prognostic marker and – more important – as an interesting immunotherapeutic target, particularly in the context of tumors with an immune exhausted and evasive TIME characterized by a poor prognosis that require intensified treatment regimens.

## Data Availability

The raw data supporting the conclusions of this article will be made available by the authors, without undue reservation.
