# Supplementary material for: HLA-G expression associates with immune evasion muscle-invasive urothelial cancer and drives prognostic relevance
Source: Front Immunol. 2024 Oct 14;15:1478196. doi: 10.3389/fimmu.2024.1478196 (PMC11513269; doi:10.3389/fimmu.2024.1478196)
Supplement: Supplementary file 1 [file DataSheet1.docx]

Supplementary Material

# Supplementary Figures and Tables

## **Supplementary Figures**
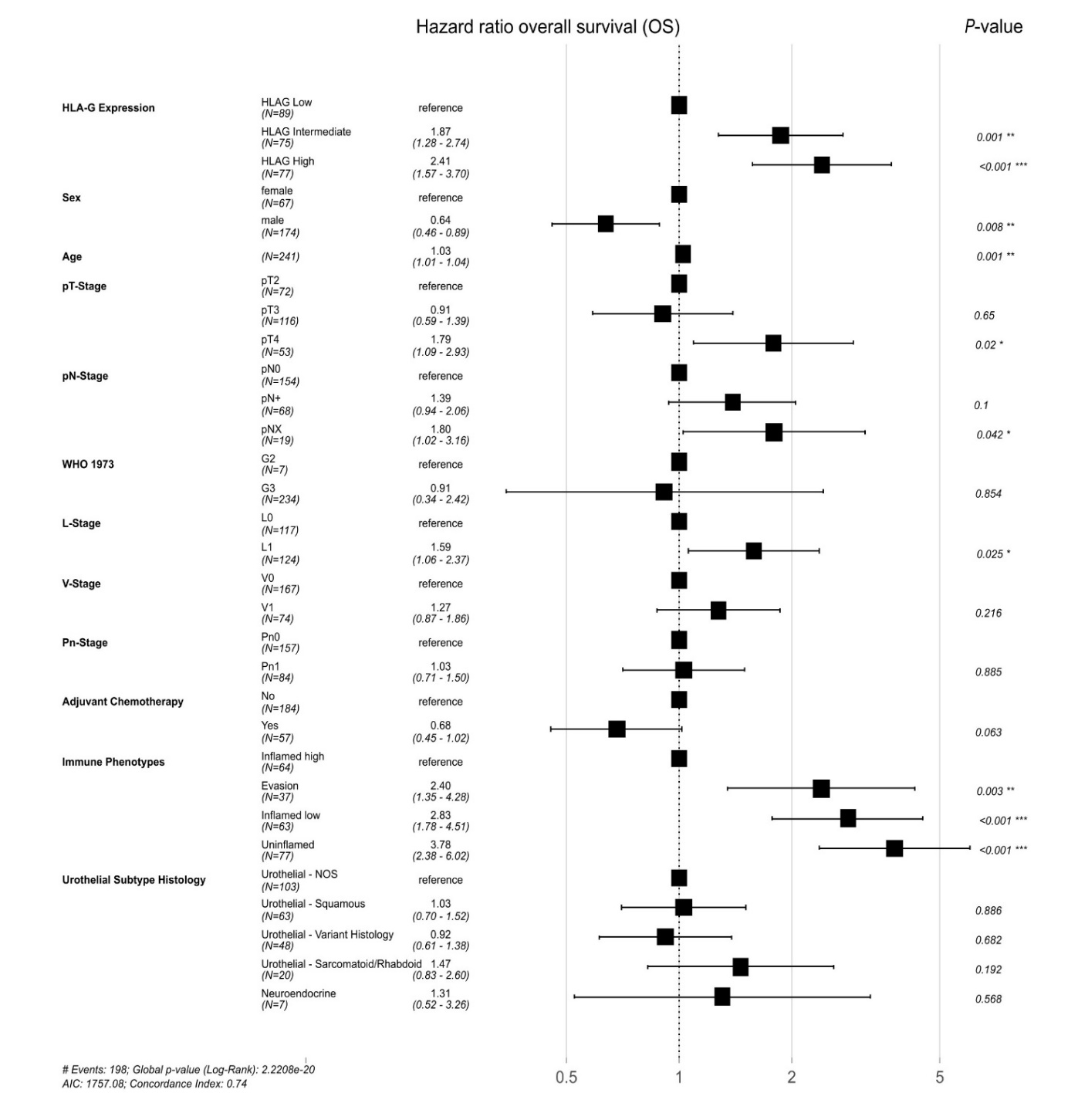
Supplementary Figure 1. Multivariable Cox-regression (survival time: unfiltered overall survival/OS; event: death by any cause) in the total cohort of n=241 patients illustrated as forest plot.


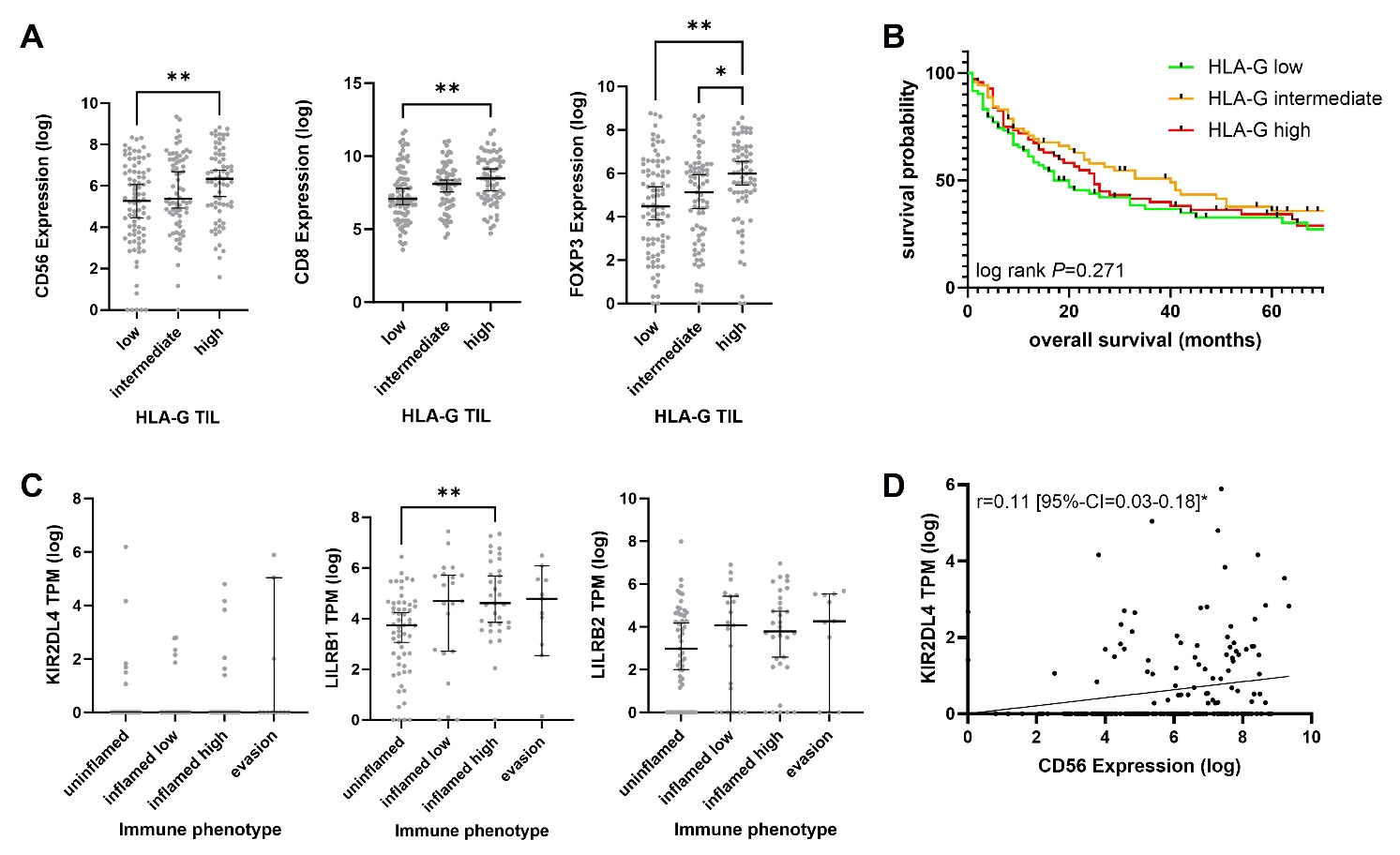


**Supplementary Figure 2 (A)** Characterization of NK cell (CD56), T cell (CD8) and T reg (FOXP3) markers in tumors with varying levels of HLA-G in TILs. The expression levels have been categorised as low, intermediate or high, with the thresholds set using a tertile split. **(B)** Kaplan-Meier analysis of OS based on HLA-G expression levels (low, intermediate, high) on TILs. Univariable log-rank *P* value is depicted in the lower left corner of the survival plots. **(C)** Characterization of HLA-G receptor mRNA levels (KIR2DL4, LILRB1, LILRB2) across immune phenotypes. P-values are derived by Dunn´s multiple comparisons test. **(D)** Spearman-rank Correlation of KIR2DL4 mRNA levels and CD56 expression.

Statistical significance at *P*<0.05; *Significance at *P<0.01*; **Significance at *P<0.001*; ***Significance at *P<0.0001*.

##
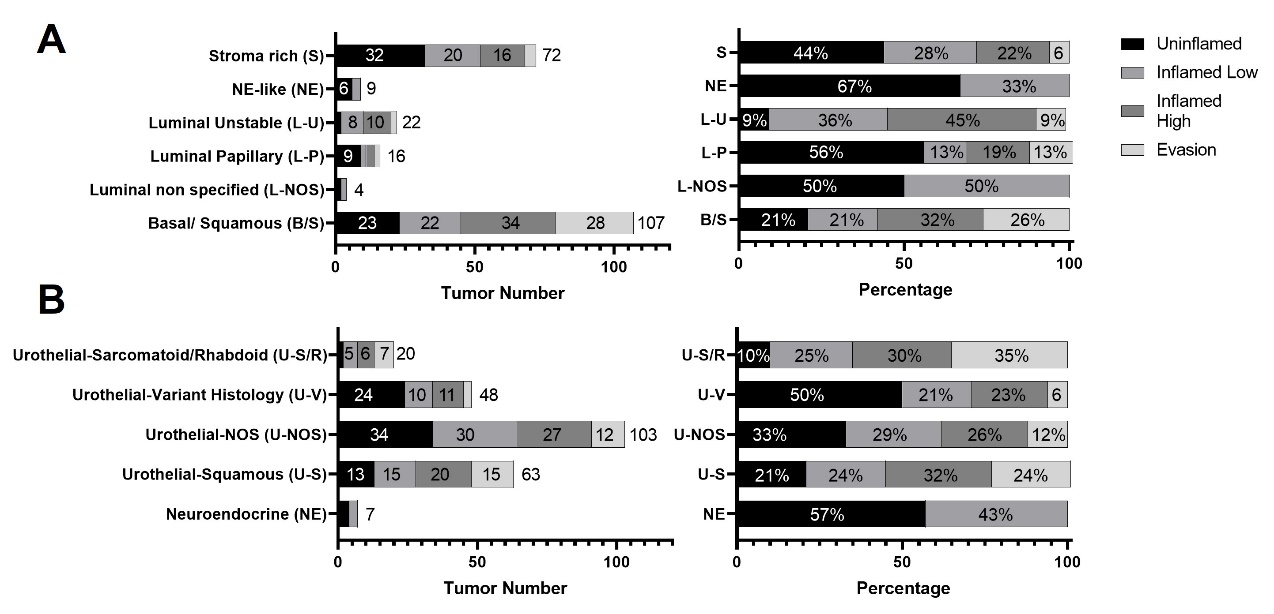


**Supplementary Figure 3.** Distribution of the immune phenotypes (uninflamed, inflamed low, inflamed high, evasion) across **(A)** urothelial subtype histology, and **(B)** molecular subtypes in absolute number and percentage.


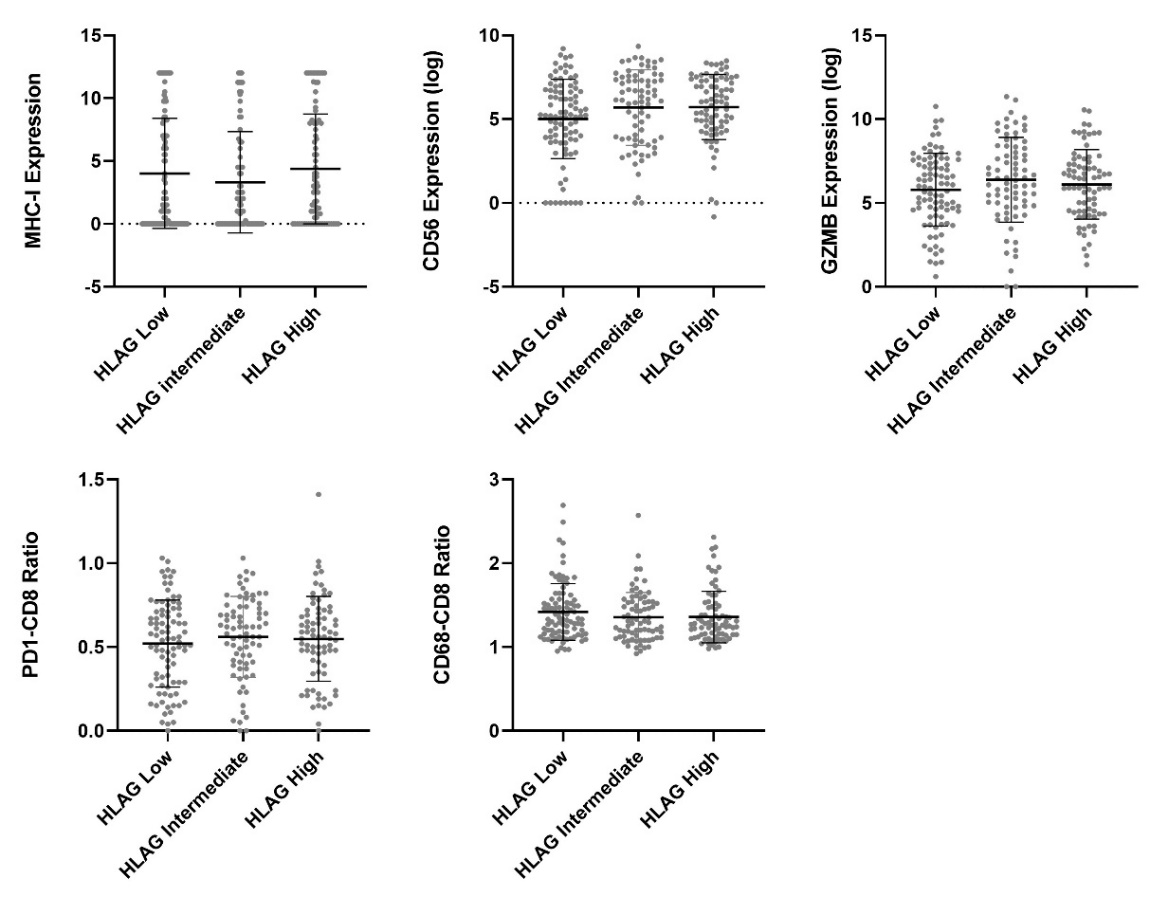


**Supplementary Figure 4.** Characterization of the TIME (MHCI, CD56, GZMB, PD1-CD8 ratio, and CD68-CD8 ratio) across HLA-G expression levels. P-values are derived by Dunn´s multiple comparisons test.


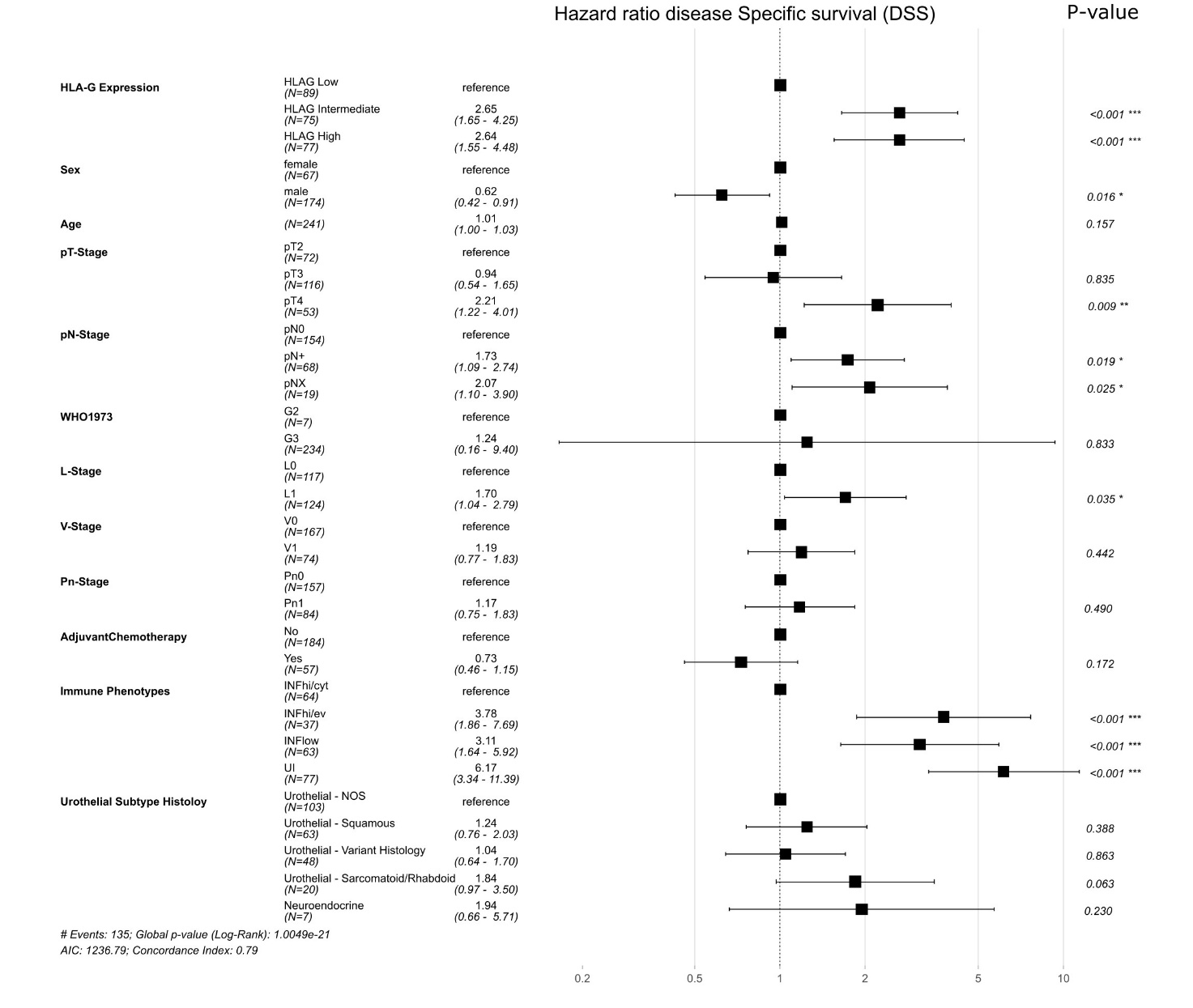


**Supplementary Figure 5.** Multivariable Cox-regression (survival time: disease specific survival/DSS; event: death by disease progression) in the total cohort of n=241 patients illustrated as forest plot.

## **Supplementary Tables**

**Supplementary Table 1.** Clinicopathological features (n=241)

**Supplementary Table 2.** Immunohistochemistry assays employed for staining of whole slide sections and tissue microarray slides.

**Supplementary Table 3A**. Shapiro- Wilk test for normal distribution (n=241)

**Supplementary Table 3B.** Kruskal-Wallis-Test and Dunn`s multiple comparisons test for HLA-G expression among urothelial subtype histology (n=241)

**Supplementary Table 3C.** Overall survival in HLA-G low/ intermediate/ high subgroup (n=241)

**Supplementary Table 3D**. Kruskal-Wallis-Test and Dunn`s multiple comparisons test for HLA-G expression among molecular subtypes (n=230)

**Supplementary Table 3E**. Kruskal-Wallis-Test and Dunn`s multiple comparisons test for HLA-G expression among immune phenotypes (n=241)

**Supplementary Table 3F.** Kruskal-Wallis-Test and Dunn`s multiple comparisons test for CD56, MHCI, CD68-CD8-ratio, PD-1-D8-ratio, and HLA-G-MHCI-ratio expression among immune phenotypes (n=241)

**Supplementary Table 4**. Correlations of HLAG vs. FOXP3 and HLAG vs. GZMB-CD8 ratio within the evasion phenotype

**Supplementary Table 5**. Kruskal-Wallis-Test and Dunn`s multiple comparisons test for CD56, MHCI, CD68-CD8-Ratio, PD-1-D8-Ratio, and HLA-G-MHCI-Ratio expression among HLA-G subgroups (n=241)
